# Association between cannabis use and physical activity in the United States based on legalization and health status

**DOI:** 10.1186/s42238-024-00248-6

**Published:** 2024-10-09

**Authors:** Ray M. Merrill, Kendyll Ashton-Hwang, Liliana Gallegos

**Affiliations:** Department of Public Health, College of Life Sciences, Brigham Young University, 2063 Life Science Building, Provo, UT 84604 USA

**Keywords:** Cannabis, Chronic medical conditions, Exercise, Marijuana, Medical cannabis, Recreational cannabis

## Abstract

**Background:**

Studies investigating the association between cannabis use and physical activity have had mixed results. This study provided a population-based assessment while determining how the relationship is affected by variables such as cannabis legalization status and chronic medical conditions.

**Methods:**

Behavior Risk Factor Surveillance System (BRFSS) data were used to evaluate the association between cannabis use and physical activity among adults ages 18 years and older in several states and territories of the U.S. during 2016–2022. Adjusted odds ratios (ORs) measuring the relationship between physical activity in the past 30 days (yes vs. no) and cannabis use in the past 30 days (yes vs. no) based on legalization and health status were estimated using logistic regression.

**Results:**

Physical activity increased from 73.16% in 2016 to 75.72% in 2022 (3.5% increase) and current cannabis use increased from 7.48% in 2016 to 14.71% in 2022 (96.7% increase). Current cannabis use was 6.5% higher in areas of legalized recreational cannabis (vs. not legal) and 0.7% higher in areas of legalized medical cannabis (vs. not legal). For the combined years, the OR measuring the association between cannabis use and physical activity was 1.24 (95% CI 1.10–1.41), after adjusting for age, sex, race/ethnicity, marital status, employment status, education, smoking status, weight classification, legal status, and chronic medical condition. The adjusted OR was 1.47 (95% CI 1.34–1.62) in areas with legalized recreational and medical cannabis (vs. illegal) and 1.05 (95% CI 0.98–1.12) in areas with legalized medical cannabis only (vs. illegal). Having a medical condition was significantly associated with lower prevalence of physical activity in the adjusted models (overall adjusted OR = 0.79, 95% CI 0.73–0.85). However, this significantly lower odds ratio was insignificant for current cannabis users.

**Conclusions:**

Public policy and personal health behaviors may improve with the findings that legal medical cannabis promotes greater physical activity in those experiencing chronic medical conditions and legal recreational cannabis promotes (even more so) greater physical activity in those not experiencing chronic medical conditions.

## Introduction

Studies investigating the association between cannabis use and physical activity have had mixed results. In research based on national longitudinal data from the Add Health Study (Wave IV, 2008–2009, *n* = 14,784, ages 24–34 and Wave V, 2016–2018, *n* = 12,043, ages 33–42), cannabis users were equal to or more likely to exercise than nonusers (French et al. 2021). A study of adults ages 18–59 years (*n* = 4,666) in the National Health and Nutrition Examination Survey (NHANES) (2011–2014) found that daily light physical activity determined using accelerometer-measured physical activity was 4% greater in individuals who used cannabis in the prior month (Xue et al. 2024). A study using accelerometer-measured physical activity in a sample of 2,092 participants ages 20–59 years from the 2005–2006 NHANES found that frequent cannabis users were more physically active than non-current users, and light cannabis users had greater odds of reporting being physically active than non-current users (Ong et al. 2021). In addition to these studies involving adults, research involving 387 adolescents ages 15–18 found that those who exercised more also reported higher cannabis use (Pacheco et al., 2021).

On the other hand, some studies have failed to find a positive association between cannabis use and physical activity. An investigation of adults from the Add Health Study (Wave V, 2016–2018, *n* = 2,591, ages 33–42) found no relationship between cannabis use and strength training or general physical activity; however, cannabis users had significantly higher weekly duration of time walking than non-cannabis users (Boutouis et al. 2024). A population-based sample of adults ages 20–59 years (*n* = 12,618) using 2008–2014 NHANES found the prevalence of moderate or vigorous physical activity was lower among cannabis users, with a negative association between increased cannabis use and time spent on physical activity (Vidot et al. 2017).

The inconsistent findings in these studies may be explained by different types of physical activity involved and different ways to measure physical activity. A follow-up study of 4,748 young Swiss men modeled different types of physical activity and found an increase in cannabis use in basic physical activity but not in sports and higher intensity exercises (Henchoz et al. 2014). However, a review article found that several athletic subgroups were more likely to use cannabis (Brisola et al. 2016). Similarly, a 2020 systematic review on cannabis use in sports determined that approximately one in four athletes reported using cannabis in the past year (Docter et al. 2020). Other reasons for the inconsistent findings include different ages studied and the changing legal landscape of cannabis.

An online survey of 605 current adult cannabis users found that 81.7% endorsed using cannabis simultaneously with exercise (YorkWilliams et al. 2019). Those who used cannabis concurrently with exercise were more likely to be younger (mean age 36.3 [SD = 14.9] vs. 43.1 [SD = 18.0]) and male. After adjusting for age and sex, those who used cannabis concurrently with exercise engaged in more minutes of aerobic and anaerobic exercise per week than those who did not. A majority also endorsed using cannabis before or after exercise, indicating that it enhanced enjoyment and recovery from exercise. Approximately half of the participants said that it increased their motivation to exercise.

With legalization of cannabis increasing in recent decades, there is greater potential for people to combine cannabis with physical activity. In 2016, cannabis use was legal for recreational and medical use in 17% of the U.S. states and territories and for medical use only in 43% of the area. In 2022, corresponding values were 41% and 36%. Hence, cannabis was legal at some level in 60% of the areas in 2016 and 77% of the areas in 2022. Consequently, the prevalence of cannabis use in adults increased from 7.48% in 2016 to 14.71% in 2022. Further, during 2016–2022 the prevalence of cannabis use was 9% higher in areas where medical cannabis only was legal and 81% higher in areas where recreational cannabis was legal (vs. not legal) (Merrill 2024). Of interest is whether the prevalence of being physically active is greater in areas with legalized cannabis for recreational and medical (vs. illegal) and medical only (vs. illegal).

Having a chronic medical condition such as arthritis may limit physical activity because of pain and other possible problems. People with arthritis may be concerned that physical activity might worsen their pain or joint damage, or they may not know what activities are safe (Wilcox et al. 2006). However, if cannabis use is perceived to help lower arthritis pain and other potential problems such as inflammation, it may correspond with increased physical activity among these patients. Indeed, recent research has found that CBD treatment can reduce pain and inflammation-causing fibroblasts in rheumatoid arthritis (Lowin et al. 2020; Frane et al. 2022). Hence, of interest is whether the association between having a chronic medical condition and physical activity is moderated by current cannabis use.

The purpose of the current study was to assess the association between current cannabis use and physical activity after adjusting for selected variables. The study also investigated whether the prevalence of physical activity was associated with the legalization status of cannabis and if the association between chronic medical conditions and physical activity was moderated by cannabis use. We hypothesize the following:

1. There is a positive association between current cannabis use and physical activity.
2. There is a higher prevalence of physical activity in areas with legalized cannabis, particularly for recreation.
3. The relationship between chronic medical conditions and physical activity is moderated by current cannabis use.

## Methods

### Data

This is a cross-sectional study that uses a standardized questionnaire to obtain data from U.S. adults 2016 through 2022. Data were obtained from the Behavior Risk Factor Surveillance System (BRFSS), which is a national system of health-related telephone surveys that collect state and territory data about U.S. residents regarding their health-related risks behaviors, chronic health conditions, and use of preventive services. The BRFSS completes over 400,000 adult surveys each year. The survey design uses random probability samples of the adult (ages 18 and older) population. The questionnaire consists of three parts: (1) core questions on demographics, current health-related conditions, and behaviors adopted by all states and U.S. territories; (2) optional modules on specific topics (e.g., marijuana or cannabis use) that states may choose to use; and (3) state-added questions developed by states for their own use (Healthy People 2030). Overall median response rates for participating areas were 47.1% in 2016, 45.9% in 2017, 49.9% in 2018, 49.4% in 2019, 47.9% in 2020, 44.0% in 2021, and 45.1% in 2022 (CDC BRFSS 2016, 2017, 2018, 2019, 2020, 2021, 2022).

Survey questions on cannabis use were added to the BRFSS in 2016 as an optional module and have been used by U.S. states and territories since. This study includes participants who were administered the cannabis use module in the 2016 through 2022 BRFSS surveys. The number of participating areas during these years are 10 states (*n* = 106,820), 10 states and 1 territory (*n* = 63,451), 13 states and 2 territories (*n* = 113,543), 12 states and 1 territory (*n* = 89,007), 20 states and 1 territory (*n* = 80,188), 20 states and 1 territory (*n* = 137,560), and 17 states and 1 territory (*n* = 94,919), respectively.

All participants provided informed consent prior to the interview. Information about the BRFSS survey design, questionnaires, and data collection is available elsewhere (CDC BRFSS 2024). This study was determined to be exempt from human subject research review by the author’s institutional review board because the BRFSS provides publicly available deidentified data.

### Measures

The primary dependent variable was adult physical activity. This variable was based on the question: “Did you participate in physical activity or exercise during the past 30 days apart from your regular job?” (CDC BRFSS 2022). The primary independent variable was current cannabis use. This variable was based on the question “During the past 30 days, on how many days did you use marijuana or cannabis?” (Azofeifa et al. 2016). Participants responded “yes” or “no” to both these questions.

Note that BRFSS treats marijuana and cannabis as synonymous. Although the cannabis plant contains about 540 chemical substances, the word marijuana typically refers to the part of or products from the plant that contain substantial amounts of tetrahydrocannabinol (THC) (NCCIH 2019; Steinmetz 2017). This study will also treat marijuana and cannabis synonymously.

Morbidity was assessed by the question, “Has a doctor, nurse, or other health professional ever told you that you had any of the following?” with answers including stroke, heart attack, coronary heart disease (CHD), asthma, chronic obstructive pulmonary disease (COPD), diabetes, arthritis, kidney disease, skin cancer, other types of cancer, and depressive disorder (CDC BRFSS 2022). A variable was created to indicate whether they had any of these chronic medical conditions.

A variable that was only available in 2018 through 2021 surveys identified the primary purpose for using cannabis, based on the question “When you used marijuana or cannabis during the past 30 days, was it usually for (1) medical reasons, (2) non-medical reasons, or (3) for both medical and non-medical reasons” (CDC BRFSS 2021). This study combines reasons 2 and 3.

Other variables included were age (18–34, 35–54, ≥ 55), sex (men, women), race/ethnicity (non-Hispanic white, non-Hispanic black, Hispanic, and other), education level (< high school, high school, some college, and college), employment status (employed, not employed, student, homemaker/retired), smoked > 100 cigarettes in lifetime (yes, no), body mass index (BMI), and legal status (recreational, medical, not legal). All areas that legalized recreational cannabis had also legalized it for medical purposes. This variable was determined by identifying for each year whether the areas participating in the cannabis module had legalized cannabis for recreational use, medical use, or neither (Forbes Health 2024). Four categories of BMI provided by the BRFSS are: underweight (BMI < 18.5 kg/m^2^), normal weight (18.5 ≤ BMI < 25.0 kg/m^2^), overweight (25.5 ≤ BMI < 30.0 kg/m^2^), and obese (≥ 30 kg/m^2^) (National Heart, Lung, and Blood Institute 2008; World Health Organization 1995).

### Statistical analysis

Prevalence of physical activity and current cannabis use were estimated by taking the survey stratum and sampling weights into consideration. Multiple logistic regression on sample survey data was used to identify whether there were associations after adjusting for age, sex, marital status, race/ethnicity, education, employment, smoking, BMI, legal status, and chronic medical conditions. Adjusted odds ratios were combined across years by taking their sample size weighted average. Variable effects were assessed for statistical significance in the model using the t test. Interactions were assessed for statistical significance using the F test. The modifying effect of legal cannabis status on cannabis use and physical activity and of cannabis use on chronic medical conditions and physical activity were assessed by comparing whether stratified ORs significantly differed from unity. Odds ratios were estimated to measure the association between variables, with corresponding 95% confidence intervals. Confidence intervals that do not overlap 1 indicate statistical significance of the odds ratio. Statistical significance was based on the 0.05 level. Statistical analyses were conducted using Statistical Analysis System (SAS) software, version 9.4 (SAS Institute Inc., Cary, NC, USA, 2014).

## Results

The prevalence of current cannabis use among adults in the U.S. increased 96.7% from 2016 (7.48% [SE = 0.17%]) through 2022 (14.71% [SE = 0.24%]), with an estimated annual percent change of 9.0% (t = 3.82, *p* = 0.0123). During these years, the prevalence of current cannabis use was 0.65% higher in areas with legalized medical cannabis only (t = 0.59, *p* = 0.5622) and 6.47% higher in areas with legalized recreational and medical cannabis (t = 5.83, t < 0.0001) versus areas where cannabis use was not legal.

**Fig. 1:**
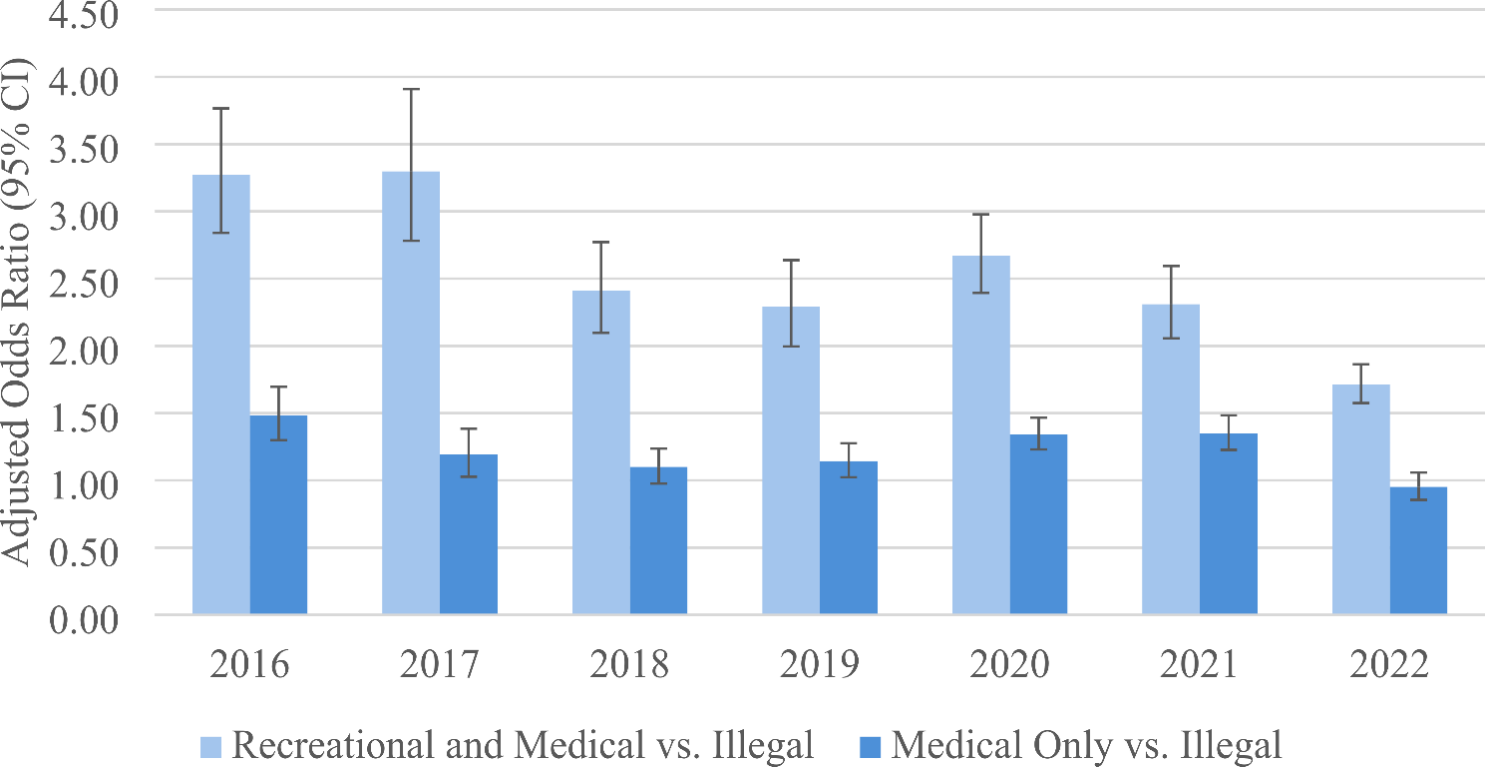
Adjusted odds of current cannabis use (yes vs. no) according to cannabis legalization status. The odds ratios are weighted and adjusted for age, sex, race, marital status, education, employment status, body mass index, tobacco use, legal status, and chronic medical conditions

The adjusted odds of current cannabis use according to selected variables is shown for the years 2016 through 2022 in Table 1. For each model, all the variables listed in the left column are statistically significant. Adjusted odds ratios (ORs) measuring the association between current cannabis use and legalization status and calendar year (shown in the table) are presented in Fig. 1. The adjusted odds of current cannabis use where cannabis is legal for recreation and medical use (vs. illegal) decrease over the study years but remains positively significant. Legalized recreational and medical cannabis has a significantly greater positive association with current cannabis use than legalized medical cannabis only. Yet, legalized medical cannabis only is also generally associated with significantly greater cannabis use.

**Table 1:** Adjusted odds of current cannabis use among adults in the U.S. by selected variables in 2016–2022

	2016	2017	2018	2019	2020	2021	2022
	OR (95% CI)^†^	OR (95% CI)^†^	OR (95% CI)^†^	OR (95% CI)^†^	OR (95% CI)^†^	OR (95% CI)^†^	OR (95% CI)^†^
Age Group							
18–34	**4.46 (3.77–5.27)**	**6.02 (4.77–7.59)**	**4.20 (3.63–4.86)**	**4.16 (3.58–4.84)**	**4.18 (3.69–4.74)**	**5.15 (4.50–5.88)**	**4.75 (4.09–5.50)**
35–54	**2.26 (1.93–2.65)**	**2.45 (1.97–3.05)**	**1.88 (1.64–2.15)**	**2.00 (1.74–2.30)**	**2.03 (1.81–2.27)**	**2.24 (1.98–2.53)**	**2.29 (2.04–2.58)**
≥ 55	1.00	1.00	1.00	1.00	1.00	1.00	1.00
Sex							
Male	**1.67 (1.49–1.87)**	**1.66 (1.41–1.95)**	**1.52 (1.37–1.68)**	**1.42 (1.28–1.57)**	**1.53 (1.40–1.66)**	**1.43 (1.31–1.55)**	**1.33 (1.22–1.45)**
Female	1.00	1.00	1.00	1.00	1.00	1.00	1.00
Race/Ethnicity							
NH White	1.00	1.00	1.00	1.00	1.00	1.00	1.00
NH Black	**1.35 (1.13–1.61)**	**1.31 (1.03–1.68)**	**1.45 (1.24–1.69)**	**1.36 (1.16–1.59)**	**1.50 (1.32–1.70)**	**1.47 (1.27–1.70)**	**1.25 (1.08–1.44)**
Hispanic	**0.75 (0.61–0.92)**	**0.56 (0.44–0.71)**	**0.78 (0.67–0.90)**	**0.62 (0.52–0.73)**	1.17 (0.94–1.45)	**0.76 (0.63–0.92)**	**0.67 (0.59–0.77)**
Other	0.90 (0.73–1.10)	**0.55 (0.42–0.74)**	**0.69 (0.56–0.84)**	**0.53 (0.43–0.65)**	0.90 (0.78–1.02)	**0.80 (0.69–0.92)**	**0.84 (0.72–0.98)**
Marital Status							
Married/Partner	1.00	1.00	1.00	1.00	1.00	1.00	1.00
Prev Married	**1.17 (1.01–1.36)**	**1.32 (1.07–1.62)**	**1.24 (1.10–1.41)**	**1.22 (1.07–1.39)**	**1.28 (1.15–1.43)**	**1.25 (1.11–1.39)**	**1.15 (1.03–1.27)**
Never Married	**1.93 (1.69–2.20)**	**1.69 (1.38–2.06)**	**1.80 (1.59–2.05)**	**1.56 (1.38–1.77)**	**1.59 (1.43–1.78)**	**1.57 (1.41–1.76)**	**1.49 (1.33–1.66)**
Unknown	1.68 (0.87–3.25)	1.19 (0.44–3.24)	0.82 (0.39–1.69)	1.17 (0.63–2.16)	0.86 (0.52–1.42)	0.66 (0.43-1.00)	1.03 (0.64–1.64)
Employment Status							
Employed	1.00	1.00	1.00	1.00	1.00	1.00	1.00
Unemployed	**1.19 (1.03–1.38)**	**1.24 (1.00-1.55)**	**1.18 (1.03–1.36)**	**1.30 (1.12–1.49)**	**1.26 (1.13–1.41)**	**1.46 (1.30–1.65)**	**1.31 (1.16–1.49)**
Student	1.08 (0.87–1.35)	0.88 (0.63–1.23)	0.90 (0.73–1.11)	0.93 (0.75–1.16)	1.00 (0.82–1.22)	**0.78 (0.63–0.96)**	0.93 (0.76–1.15)
Homemaker/Retired	**0.56 (0.47–0.67)**	**0.59 (0.46–0.76)**	**0.62 (0.53–0.73)**	**0.67 (0.57–0.79)**	**0.63 (0.54–0.72)**	**0.61 (0.53–0.70)**	**0.72 (0.63–0.82)**
Other	**1.21 (1.06–1.38)**	**1.35 (1.11–1.64)**	**1.16 (1.03–1.32)**	**0.41 (0.20–0.82)**	1.04 (0.67–1.63)	0.87 (0.57–1.32)	0.74 (0.49–1.11)
Education							
< High School	1.01 (0.83–1.22)	0.86 (0.63–1.17)	0.91 (0.76–1.09)	**0.69 (0.57–0.83)**	**1.19 (1.01–1.41)**	0.97 (0.81–1.17)	0.94 (0.79–1.13)
High School	1.00	1.00	1.00	1.00	1.00	1.00	1.00
Some Coll/Tech	**1.21 (1.06–1.38)**	**1.35 (1.11–1.64)**	**1.16 (1.03–1.32)**	**1.29 (1.13–1.46)**	**1.10 (1.00-1.22)**	1.08 (0.97–1.20)	1.08 (0.97–1.19)
Completed Coll/Tech	0.91 (0.79–1.05)	0.98 (0.81–1.19)	0.97 (0.86–1.10)	0.92 (0.81–1.05)	0.98 (0.88–1.08)	**0.87 (0.79–0.97)**	**0.88 (0.80–0.98)**
Unknown	1.08 (0.30–3.82)	**0.15 (0.04–0.60)**	**0.24 (0.06–0.96)**	**0.10 (0.02–0.49)**	0.44 (0.18–1.05)	1.62 (0.69–3.81)	0.36 (0.13–1.01)
Smoked ≥ 100 Cigs Life							
Yes	**3.84 (3.41–4.33)**	**3.55 (3.02–4.17)**	**3.30 (2.98–3.67)**	**2.90 (2.61–3.23)**	**3.62 (3.31–3.97)**	**3.25 (2.97–3.55)**	**3.38 (3.09–3.69)**
No	1.00	1.00	1.00	1.00	1.00	1.00	1.00
Unknown	**2.47 (1.17–5.19)**	1.71 (0.55–5.31)	0.80 (0.37–1.75)	1.45 (0.75–2.83)	**2.81 (1.58-5.00)**	**2.23 (1.26–3.95)**	**2.28 (1.50–3.48)**
Weight Classification							
Underweight	0.83 (0.60–1.15)	0.95 (0.62–1.45)	1.06 (0.78–1.43)	0.99 (0.72–1.37)	1.15 (0.90–1.48)	1.13 (0.86–1.50)	1.19 (0.89–1.59)
Normal weight	1.00	1.00	1.00	1.00	1.00	1.00	1.00
Overweight	**0.70 (0.61–0.79)**	**0.65 (0.55–0.78)**	**0.78 (0.69–0.87)**	**0.74 (0.66–0.83)**	**0.72 (0.65–0.79)**	**0.76 (0.69–0.85)**	**0.79 (0.71–0.87)**
Obese	**0.54 (0.47–0.61)**	**0.49 (0.40–0.60)**	**0.62 (0.55–0.71)**	**0.65 (0.58–0.74)**	**0.63 (0.56–0.70)**	**0.64 (0.57–0.71)**	**0.62 (0.56–0.69)**
Legal Status							
Illegal	1.00	1.00	1.00	1.00	1.00	1.00	1.00
Medical Only	**1.49 (1.30–1.70)**	**1.19 (1.03–1.39)**	1.10 (0.98–1.24)	**1.14 (1.02–1.28)**	**1.23 (1.13–1.34)**	**1.35 (1.23–1.48)**	**0.95 (0.86–1.06)**
Med and Recreational	**3.27 (2.84–3.77)**	**3.30 (2.78–3.91)**	**2.41 (2.10–2.77)**	**2.29 (2.00-2.64)**	**1.60 (1.37–1.87)**	**2.31 (2.06–2.60)**	**1.71 (1.58–1.86)**
Chronic Med Condition							
No	1.00	1.00	1.00	1.00	1.00	1.00	1.00
Yes	**1.36 (1.21–1.53)**	**1.60 (1.35–1.90)**	**1.60 (1.44–1.78)**	**1.55 (1.39–1.73)**	**1.66 (1.51–1.83)**	**1.71 (1.56–1.88)**	**1.70 (1.55–1.87)**

^†^Weighted odds ratios (ORs) and 95% confidence intervals (CIs). For each year, the ORs are adjusted for all the variables listed in the left column. Bolded odds ratios are statistically significant at the 0.05 level

The odds of current cannabis use is significantly greater among those with a chronic medical condition, in each year (Table 1). The adjusted ORs significantly increased from 1.36 (95% CI 1.21–1.53) in 2016 to 1.70 (95% CI 1.55–1.87) in 2022.

### Hypothesis 1. There is a positive association between current cannabis use and physical activity

**Fig. 2:**
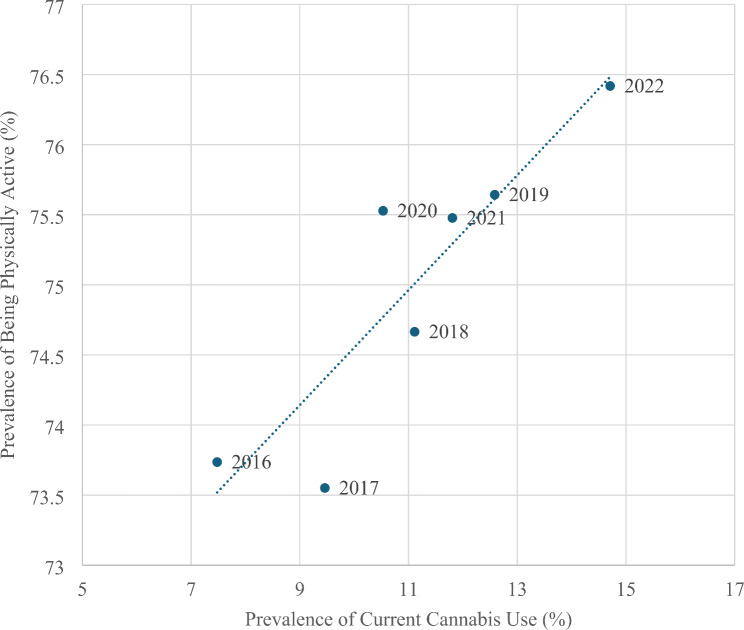
Correlation between being physically active (yes vs. no) and current cannabis use in adults during the years 2016–2022

**Fig. 3:**
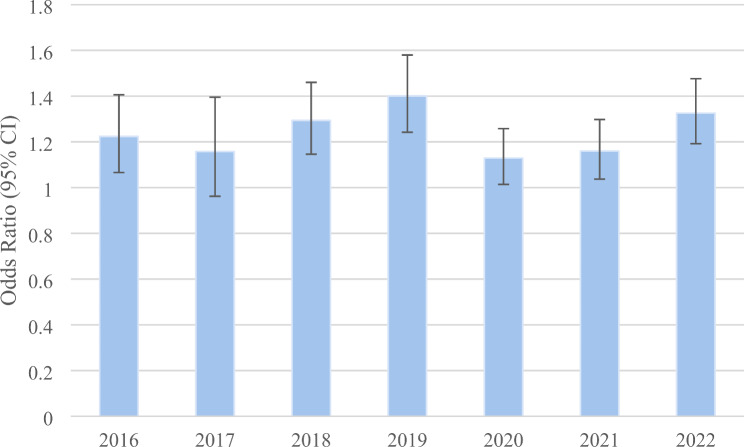
Adjusted odds of physical activity (yes vs. no) among current cannabis users versus non-users. The odds ratios are weighted and adjusted for age, sex, race, marital status, education, employment status, tobacco smoking status, body mass index (BMI), legal status, and chronic medical conditions

The prevalence of adults who are physically active in the U.S. increased 3.5% from 2016 (73.16% [SE = 0.29%]) to 2022 (75.72% [SE = 0.24%]), with an estimated annual percent change of 0.6% (t = 5.59, *p* = 0.0025). A positive linear association exists between the prevalence of current cannabis use and the prevalence of being physically active over the study period (*r* = 0.789, t = 2.88, *p* = 0.0348) (Fig. 2). Adjusted ORs measuring the association between current cannabis use and physical activity are significantly positive across the study years (except in 2017, where it is marginally insignificant) (Table 2). These adjusted ORs are presented in Fig. 3, with their weighted average across the years of 1.24 (95% CI 1.10–1.41). All other variables in the table are also significantly associated with being physically active.

**Table 2:** Adjusted odds of physical activity (yes vs. no) among adults in the U.S. by cannabis status and other variables in 2016–2022

	2016	2017	2018	2019	2020	2021	2022
	OR (95% CI)^†^	OR (95% CI)^†^	OR (95% CI)^†^	OR (95% CI)^†^	OR (95% CI)^†^	OR (95% CI)^†^	OR (95% CI)^†^
Cannabis use (≤ 30 days)							
No	1.00	1.00	1.00	1.00	1.00	1.00	1.00
Yes	**1.22 (1.07–1.41)**	1.16 (0.96–1.40)	**1.29 (1.15–1.46)**	**1.40 (1.24–1.58)**	**1.14 (1.02–1.27)**	**1.16 (1.04–1.30)**	**1.33 (1.19–1.48)**
Unknown	0.94 (0.63–1.41)	1.20 (0.79–1.82)	0.88 (0.59–1.32)	1.22 (0.82–1.80)	1.01 (0.75–1.36)	1.02 (0.78–1.34)	1.07 (0.83–1.38)
Age Group							
18–34	**1.80 (1.61–2.02)**	**1.40 (1.22–1.62)**	**1.62 (1.45–1.81)**	**1.25 (1.11–1.40)**	**1.61 (1.45–1.78)**	**1.63 (1.46–1.81)**	**1.53 (1.36–1.72)**
35–54	**1.35 (1.24–1.46)**	**1.18 (1.05–1.31)**	**1.25 (1.15–1.36)**	**1.19 (1.09–1.30)**	**1.25 (1.16–1.35)**	**1.40 (1.29–1.52)**	**1.32 (1.21–1.44)**
≥ 55	1.00	1.00	1.00	1.00	1.00	1.00	1.00
Sex							
Male	**1.25 (1.18–1.34)**	**1.19 (1.09–1.31)**	**1.26 (1.18–1.35)**	**1.16 (1.07–1.24)**	**1.17 (1.11–1.25)**	**1.23 (1.16–1.31)**	**1.30 (1.21–1.39)**
Female	1.00	1.00	1.00	1.00	1.00	1.00	1.00
Race/Ethnicity							
NH White	1.00	1.00	1.00	1.00	1.00	1.00	1.00
NH Black	**0.72 (0.64–0.81)**	**0.80 (0.69–0.92)**	**0.85 (0.76–0.95)**	**0.78 (0.70–0.87)**	**0.78 (0.71–0.85)**	**0.79 (0.69–0.90)**	**0.88 (0.78–0.98)**
Hispanic	**0.55 (0.49–0.62)**	**0.51 (0.46–0.58)**	**0.61 (0.55–0.67)**	**0.74 (0.66–0.83)**	**0.72 (0.60–0.87)**	**0.69 (0.59–0.80)**	**0.86 (0.77–0.96)**
Other	**0.80 (0.69–0.93)**	**0.80 (0.65–0.99)**	**0.72 (0.61–0.86)**	**0.80 (0.68–0.95)**	**0.79 (0.69–0.89)**	**0.81 (0.73–0.91)**	**0.82 (0.72–0.94)**
Marital Status							
Married/Partner	1.00	1.00	1.00	1.00	1.00	1.00	1.00
Prev Married	**0.80 (0.75–0.86)**	**0.88 (0.80–0.97)**	**0.71 (0.66–0.77)**	**0.79 (0.72–0.85)**	**0.72 (0.67–0.77)**	**0.76 (0.70–0.81)**	**0.75 (0.69–0.81)**
Never Married	**0.94 (0.84–1.05)**	1.02 (0.89–1.17)	**0.83 (0.75–0.91)**	0.92 (0.82–1.02)	**0.89 (0.81–0.97)**	**0.81 (0.74–0.90)**	0.90 (0.81-1.00)
Unknown	1.10 (0.71–1.71)	0.75 (0.39–1.47)	1.39 (0.88–2.21)	1.00 (0.56–1.79)	0.82 (0.53–1.26)	1.00 (0.59–1.69)	0.84 (0.57–1.25)
Employment Status							
Employed	1.00	1.00	1.00	1.00	1.00	1.00	1.00
Unemployed	**0.51 (0.46–0.56)**	**0.72 (0.63–0.82)**	**0.57 (0.51–0.62)**	**0.64 (0.58–0.72)**	**0.58 (0.53–0.63)**	**0.62 (0.56–0.69)**	**0.46 (0.42–0.52)**
Student	**1.65 (1.30–2.08)**	1.29 (0.98–1.70)	1.11 (0.89–1.39)	**1.46 (1.17–1.83)**	1.18 (0.95–1.48)	1.04 (0.83–1.32)	**1.36 (1.07–1.73)**
Homemaker/Retired	**0.91 (0.84–0.99)**	1.09 (0.97–1.22)	0.95 (0.87–1.04)	**1.10 (1.00-1.20)**	**0.86 (0.79–0.92)**	**0.89 (0.82–0.97)**	**0.89 (0.81–0.97)**
Other	0.72 (0.45–1.15)	0.97 (0.54–1.76)	1.10 (0.73–1.65)	0.97 (0.56–1.70)	0.86 (0.62–1.20)	0.85 (0.61–1.19)	**0.70 (0.53–0.92)**
Education							
< High School	**0.64 (0.58–0.72)**	**0.81 (0.71–0.92)**	**0.76 (0.68–0.84)**	**0.67 (0.59–0.75)**	**0.84 (0.74–0.94)**	**0.71 (0.61–0.81)**	**0.66 (0.58–0.75)**
High School	1.00	1.00	1.00	1.00	1.00	1.00	1.00
Some Coll/Tech	**1.50 (1.39–1.61)**	**1.33 (1.20–1.49)**	**1.47 (1.35–1.60)**	**1.46 (1.33–1.59)**	**1.54 (1.44–1.66)**	**1.46 (1.36–1.57)**	**1.46 (1.34–1.59)**
Completed Coll/Tech	**2.80 (2.59–3.03)**	**2.07 (1.86–2.31)**	**2.31 (2.11–2.53)**	**2.35 (2.14–2.59)**	**2.63 (2.44–2.83)**	**2.51 (2.32–2.71)**	**2.46 (2.26–2.69)**
Unknown	1.40 (0.77–2.54)	0.69 (0.26–1.86)	0.98 (0.47–2.03)	0.87 (0.34–2.25)	**2.00 (1.26–3.18)**	0.85 (0.29–2.48)	0.89 (0.53–1.51)
Smoked ≥ 100 Cigs Life							
Yes	**0.84 (0.79–0.90)**	**0.81 (0.75–0.89)**	**0.81 (0.76–0.87)**	**0.85 (0.79–0.92)**	**0.82 (0.77–0.87)**	**0.78 (0.73–0.84)**	**0.85 (0.79–0.91)**
No	1.00	1.00	1.00	1.00	1.00	1.00	1.00
Unknown	**0.64 (0.42–0.99)**	0.93 (0.55–1.60)	0.83 (0.52–1.34)	1.17 (0.72–1.89)	0.76 (0.49–1.18)	0.97 (0.69–1.36)	0.74 (0.53–1.04)
Weight Classification							
Underweight	0.86 (0.68–1.09)	**0.45 (0.30–0.66)**	**0.51 (0.40–0.64)**	**0.71 (0.53–0.95)**	**0.63 (0.52–0.77)**	**0.67 (0.52–0.87)**	**0.64 (0.50–0.82)**
Normal weight	1.00	1.00	1.00	1.00	1.00	1.00	1.00
Overweight	0.93 (0.86–1.01)	**0.85 (0.76–0.95)**	**0.85 (0.78–0.93)**	0.95 (0.86–1.04)	0.94 (0.87–1.02)	0.94 (0.86–1.02)	**0.90 (0.82–0.99)**
Obese	**0.66 (0.61–0.71)**	**0.63 (0.57–0.70)**	**0.60 (0.55–0.65)**	**0.63 (0.58–0.69)**	**0.60 (0.56–0.65)**	**0.59 (0.55–0.64)**	**0.60 (0.55–0.66)**
Legal Status							
Illegal	1.00	1.00	1.00	1.00	1.00	1.00	1.00
Medical Only	0.94 (0.88–1.01)	**0.88 (0.82–0.95)**	1.04 (0.97–1.11)	**1.16 (1.08–1.24)**	**1.14 (1.08–1.20)**	**1.14 (1.07–1.21)**	0.96 (0.89–1.04)
Med and Recreational	**1.63 (1.49–1.78)**	**1.98 (1.77–2.21)**	**1.49 (1.34–1.65)**	**1.38 (1.25–1.53)**	1.02 (0.90–1.14)	1.00 (0.91–1.10)	**1.13 (1.06–1.21)**
Chronic Med Condition							
No	1.00	1.00	1.00	1.00	1.00	1.00	1.00
Yes	**0.78 (0.73–0.84)**	**0.80 (0.73–0.88)**	**0.83 (0.77–0.90)**	**0.86 (0.80–0.93)**	**0.72 (0.67–0.77)**	**0.74 (0.69–0.80)**	**0.80 (0.74–0.86)**

^†^Weighted odds ratios (ORs) and 95% confidence intervals (CIs). For each year, the ORs are adjusted for all the variables listed in the left column. Bolded odds ratios are statistically significant at the 0.05 level

### Hypothesis 2. There is a higher prevalence of physical activity in areas with legalized cannabis, particularly for recreation

In each year, the odds of physical activity is significantly greater in areas with legalized cannabis for recreational and medical uses (vs. illegal), except in 2021, and the odds of physical activity is significantly greater in areas with legalized cannabis for medical use only (vs. illegal) in 2019, 2020, and 2021 (Table 2). The weighted average across the years of the adjusted OR is 1.47 (95% CI 1.34–1.62) for areas with legalized recreational and medical cannabis (vs. illegal) and 1.05 (95% CI 0.98–1.12) for areas with legalized medical cannabis only (vs. illegal).

There was a tendency for the adjusted ORs between current cannabis use and physical activity to depend on legal status, with significant interactions in the years 2016, 2019, and 2021 (F *p* < 0.05). The weighted average of the adjusted OR across all years showed no significant association for illegal (1.16, 95% CI 0.93–1.44), but significant positive associations for legal medical only (1.19, 95% CI 1.02–1.40) and legal recreational and medical (1.37, 95% CI 1.09–1.73).

### Hypothesis 3. The relationship between chronic medical conditions and physical activity is moderated by cannabis use

**Fig. 4:**
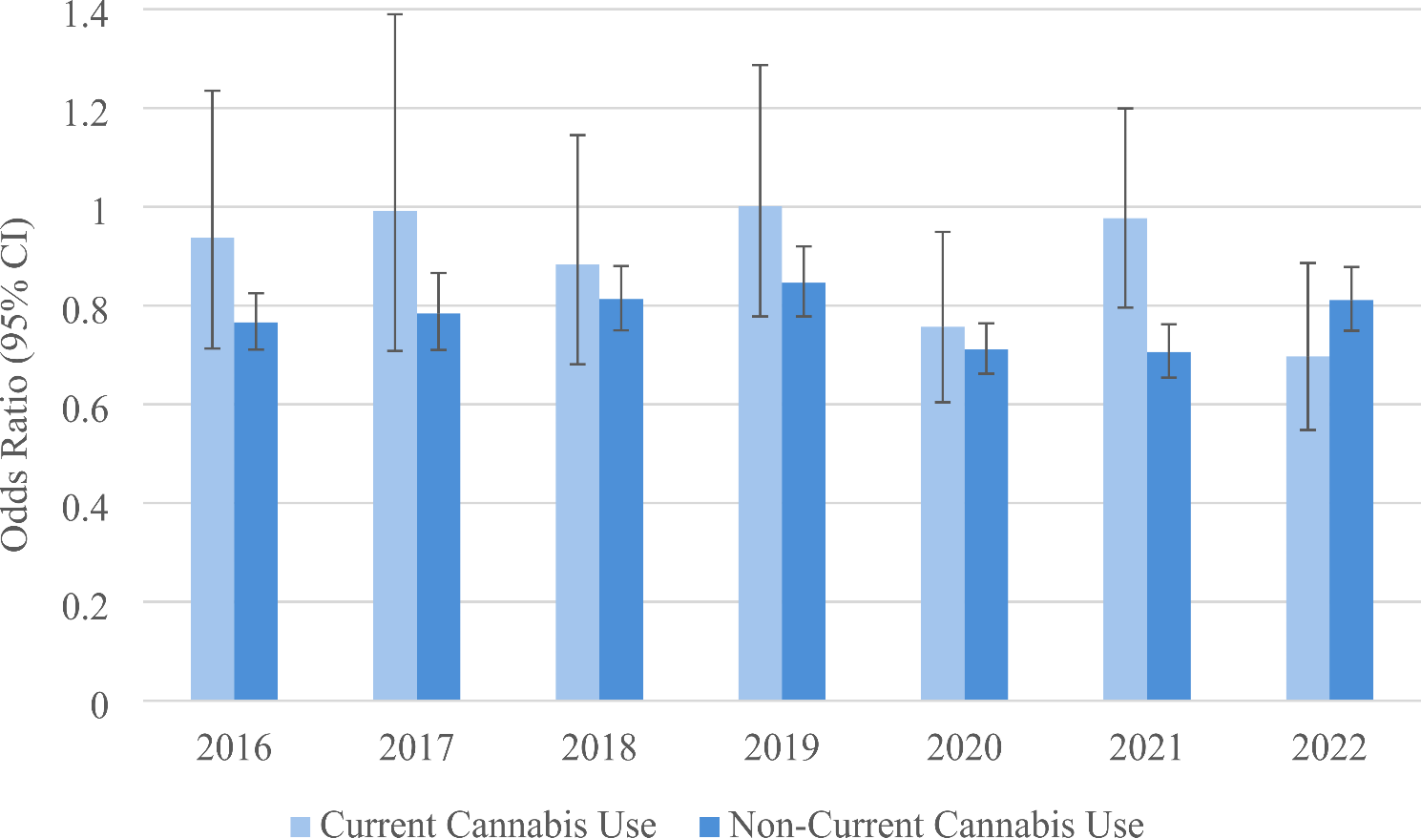
Adjusted odds ratios of physical activity according to having a medical condition (yes vs. no) across calendar years, stratified by current cannabis use status. The odds ratios are weighted and adjusted for age, sex, race, marital status, education, employment status, body mass index, tobacco use, and legal status

In each year, the odds of physical activity is consistently significantly lower in those with a chronic medical condition. The weighted average across the years of the adjusted OR is 0.79 (95% CI 0.73–0.85). The adjusted odds of physical activity by having a chronic medical condition is shown according to cannabis use status in Fig. 4. For current cannabis users, the weighted average across the years of the adjusted OR is 0.89 (95% CI 0.69–1.16), which is not statistically significant. For non-current cannabis users, the weighted average across the years of the adjusted OR is 0.78 (05% CI 0.72–0.84), which is statistically significant. However, only in 2021 is the statistical interaction involving current cannabis use and chronic medical conditions significant (F = 6.02, *p* = 0.0023).

**Fig. 5:**
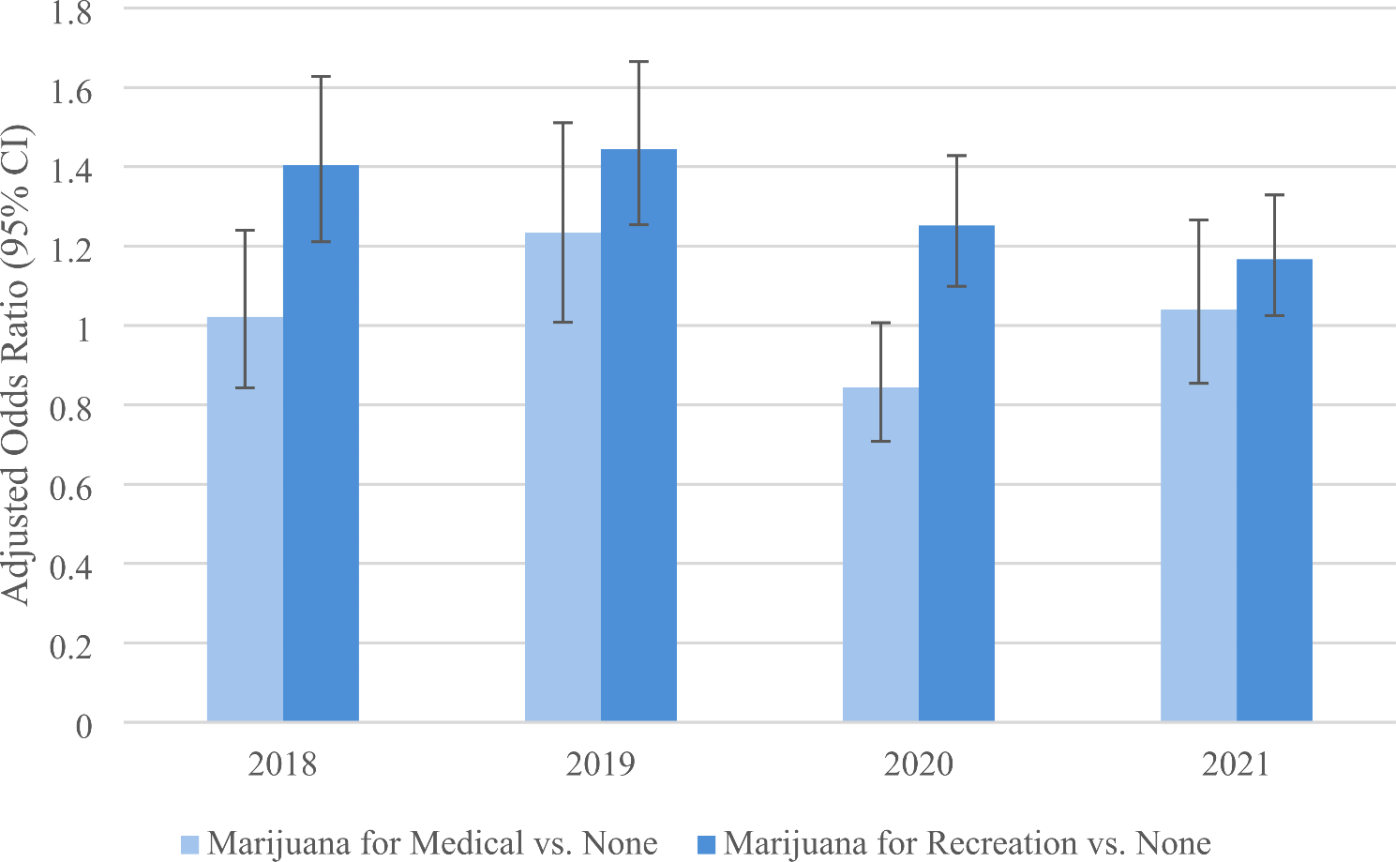
Adjusted odds of physical activity according to reason for cannabis use. The odds ratios are weighted and adjusted for age, sex, race, marital status, education, employment status, body mass index (BMI), tobacco smoking, and cannabis use. Note that BRFSS only included the question about reasons for cannabis use in the years 2018 through 2021

In the years 2018 through 2021, participants in several BRFSS areas were asked the primary reason why they used cannabis. Physical activity was not generally associated with cannabis use for medical reasons (Fig. 5). However, physical activity was significantly positively associated with cannabis use for recreational reasons.

## Discussion

This study described the increasing prevalence of current cannabis use in U.S. adults from 2016 to 2022 according to legalization status, chronic medical conditions, and other variables. Current cannabis use was significantly greater in areas where cannabis was legal, especially for recreational use. The ORs measuring the association between current cannabis use and legalization for medical and recreational use versus illegal remained significantly positive throughout the study period but decreased over time. This decrease is because cannabis use also significantly increased in areas where it was illegal, about 11% per year (t = 3.72, *p* = 0.0137), which is consistent with growing acceptance of the drug in general. The positive association between chronic disease and cannabis use significantly increased as legalization of cannabis increased. That is, an increasing proportion of people were using medical cannabis.

The primary purpose of the study was to evaluate the association between current cannabis use and physical activity, the association between legalization of cannabis and physical activity, and if the association between chronic medical conditions and physical activity were moderated by cannabis use.

### Hypothesis 1. There is a positive association between current cannabis use and physical activity

A positive association between current cannabis use and physical activity was observed after adjusting for selected demographic variables, smoking, BMI, legalization status, and chronic medical conditions. The positive association for the years 2016 through 2022 was significant (weighted adjusted OR = 1.24 [95% CI 1.10–1.41]), as consistent with some studies (Xue et al. 2024; Pacheco et al. 2021; Ong et al. 2021; French et al. 2021) but not others (Boutouis et al. 2024; Vidot et al. 2017). Possible reasons why some studies have not found a positive association between current cannabis use and physical activity while we and others have may be due to differences in the types of physical activity involved, ways physical activity is measured, different ages evaluated, and changes in the legal landscape of cannabis.

### Hypothesis 2. There is a higher prevalence of physical activity in areas with legalized cannabis, particularly for recreation

Legalization of cannabis directly corresponded to greater physical activity, especially with legalized recreational cannabis. The weighted average adjusted ORs measuring the association between current cannabis use and physical activity showed no association in illegal areas, but significantly positive association in areas with legal cannabis, more so for legal recreational cannabis.

As cannabis becomes increasingly accessible through legalization, there is greater potential for people to use it to influence their physical activity, particularly if the drug does not require authorization from a healthcare prover and the state’s approval for a specific medical purpose. In other words, it may be that the greater flexibility in how a person can use cannabis because of legalization for recreation can explain this result. Perhaps less restricted control of cannabis use increases the potential for people to identify possible benefits of combining cannabis with physical activity. In a study involving 131 adult cannabis users ages 18–55 years who completed an anonymous online survey, primary reasons for using cannabis before exercise were to help them focus (66%), enjoy the exercise experience (65%), and enhance the mind-body-spirit connection (65%) (Ogle et al. 2022). Further, a review article found that cannabidiol (CBD) may aid athletes with recovery by improving sleep quality and lowering pain and mild traumatic brain injury (Burr et al. 2021). In addition, this result is consistent with the finding that participants do not generally associate physical activity with cannabis use for medical reasons but for recreational reasons (see Fig. 5).

### Hypothesis 3. The relationship between chronic medical conditions and physical activity is moderated by cannabis use

The results show that chronic medical conditions significantly lower physical activity, which is well established (National Institute on Aging 2020). This negative association is a concern because physical inactivity is a major risk factor for many chronic conditions (Booth et al. 2012; Anderson and Durstine 2019). However, the negative association between chronic medical conditions and physical activity was only observed in non-cannabis users. In cannabis users, there was not significantly lower physical activity. Hence, there appears to be some benefits associated with cannabis use for those with chronic medical conditions that allow them to be more physically active, possibly because cannabis helps control pain and inflammation (Lowin et al. 2020; Frane et al. 2022).

In the current study, having a chronic medical condition positively associated with current cannabis use (weighted average adjusted OR = 1.60, 95% CI 1.44–1.78). Another study likewise found that adults with medical conditions were significantly more likely to use cannabis (Dai and Richter 2019). In a study of adults ages ≥ 18 years (*n* = 214,505) from the 2015–2019 National Survey on Drug Use and Health researchers specifically showed increased use of cannabis among those with difficulty hearing, walking, with 2–3 impairments, and kidney disease (Yang et al. 2023).

The positive association between chronic medical conditions and current cannabis use is consistent with research showing potential benefits of cannabis use for medical purposes. For example, because of CBD’s (the typical component of cannabis for medical use) anti-inflammatory and analgesic properties (Atalay et al. 2019; Sklenárová et al. 2023) and rising patient interest (Failing et al. 2021), studies are being performed to see its potential impact on relieving arthritic symptoms. Preliminary studies performed on animals have shown positive results with use of CBD reducing pain, decreasing inflammatory cytokine production, and increasing activity (Lima et al. 2022; Verrico et al. 2020; Grogan et al. 2023). Human studies have shown mixed yet positive results (Lowin et al. 2020; Frane et al. 2022; Vela et al. 2021). More research is necessary to fully explore this potential benefit of CBD use in arthritis patients.

People may use cannabis for treating asthma because it is an immediate bronchodilator, which relaxes the airways and helps breathing (Jarjou’i and Izbicki 2020). However, inhaled cannabis can increase cough, sputum, wheezing, and COPD and should not be smoked or vaped (Aldington et al. 2007; Tan et al. 2009).

Cannabis might benefit diabetes patients because CBD can reduce chronic inflammation. Chronic inflammation is simultaneously a risk factor and symptom of types I and II diabetes (Rissetto 2021). This chronic inflammation can damage important insulin producing cells in the pancreas (Tsalamandris et al. 2019), which further exacerbates diabetes symptoms. CBD is known to have anti-inflammatory properties (Atalay et al. 2019; Sklenárová et al. 2023) potentially valuable in treating and delaying diabetes symptoms. Studies performed have found significantly lowered blood glucose levels and increased plasma insulin levels with use of CBD products (Ehud et al. 2012; Weiss et al. 2006; Romero-Zerbo et al. 2020; Chaves et al. 2020). Also, studies found decreased apoptosis and destruction of pancreatic islet and beta cells, which increase insulin production, with the use of CBD products (Ehud et al. 2012; Romero-Zerbo et al. 2020).

There is no evidence that cannabis directly causes depression but people who use cannabis might do so to detach from their depression. Alternatively, CBD may be used to help treat depression. CBD has been shown to reduce depressive symptoms in both animal and human studies (Wright et al. 2020). While positive impacts are largely seen, researchers agree that more study into proper dosage and administration timelines should be performed to understand the true impacts (Wright et al. 2020; Shannon et al. 2019; Blessing et al. 2015; Skelley et al. 2020) Additionally, existing studies are mainly short-term evaluations. Further research is needed to explore potential consequences of long-term CBD use (Hasbi et al. 2023). It is important that psychiatrists and physicians are aware of this growing popularity regarding self-treatment with CBD. A 2022 exploratory study found that only 19% of respondents consulted with doctors and/or pharmacists regarding CBD use safety and only 49% told their psychiatrists about their CBD use (Wieckiewicz et al. 2022).

## Limitations

This study is limited in certain ways. First, BRFSS provides observational cross-sectional data. Hence, causal relationships between cannabis and physical activity cannot be established. Second, the chronic medical condition variable reflected whether a person had ever experienced one or more of 11 chronic diseases but did not consider whether the diseases were transient or persistent. Also, BRFSS does not ask about whether medical conditions are controlled or whether they are adherent with medications. Third, cannabis use, information used to compute BMI, and chronic health conditions are self-reported and prone to social desirability bias. Yet, a systematic review of publications assessing the reliability and validity of BRFSS data found that BRFSS prevalence rates were like other national surveys involving self-reports (Pierannunzi et al. 2013). Medical conditions and smoking status relied on questions regarding lifetime status and did not provide current information about the condition’s status. In addition, the physical activity variable was based on self-reported response to whether they participated in physical activity or exercise in the past 30 days other than their regular job. Unfortunately, BRFSS does not categorize physical activity based on WHO guidelines on metabolic equivalents (METs) for various types of activities or measure hours/minutes of physical activity per week or physical intensity. Fourth, the BRFSS did not provide information on the part of the cannabis plant variety primarily used (e.g., THC, CBD, CBN). While we defined current cannabis use as within the past 30 days, we did not consider the intensity of use, or the method of consumption because the BRFSS survey does not collect this information.

## Conclusion

In the U.S. adult population, current cannabis use is significantly associated with higher prevalence of physical activity. The prevalence of physical activity is significantly greater in U.S. states and territories where cannabis is legalized for recreational and medical purposes (vs. not legal). The association between current cannabis use and physical activity is not significant in areas where cannabis is illegal, but significantly positive in areas where it is legal, more so for legal recreational cannabis. When asked the primary reason for using cannabis, physical activity was not generally associated with cannabis use for medical reasons but for recreational reasons. Where cannabis was used to improve the physical activity experience, it may be to improve their focus and enjoyment, or to enhance the mind-body-spirit connection and improve recovery by improving sleep quality and lowering pain. Finally, lower physical activity among those people with chronic medical conditions may be ameliorated in some cases by cannabis use.

As public health policy strives to influence better population health from scientific knowledge about the health challenges and benefits of cannabis use, the results of this study indicate that legal medical cannabis promotes greater physical activity in those experiencing chronic medical conditions and legal recreational cannabis promotes (even more so) greater physical activity in those not experiencing chronic medical conditions.

Future studies can establish temporal or causal relationships by using longitudinal data. Qualitative assessment can also add to our understanding of how and why individuals with various types of chronic medical conditions use cannabis. The influence of chronic medical conditions on physical activity can be made more precise with the addition of information about the transient or persistent nature of the condition, and whether medication use is helping. In addition, direct measures of physical activity and cannabis use, along with the type of and method of consumption of cannabis, should be considered in future studies.

## Data Availability

No datasets were generated or analysed during the current study.

## Abbreviations

BMI: Body mass index
BRFSS: Behavior Risk Factor Surveillance System
CBD: Cannabidiol
CBN: Cannabinol
CDC: Center for Disease Control and Prevention
CHD: Coronary heart disease
CI: Confidence interval
COPD: Chronic obstructive pulmonary disease
SE: Standard Error
THC: Tetrahydrocannabinol
U.S.: United States
